# Anti-malarial activity and toxicity assessment of *Himatanthus articulatus*, a plant used to treat malaria in the Brazilian Amazon

**DOI:** 10.1186/s12936-015-0643-1

**Published:** 2015-03-27

**Authors:** Valdicley V Vale, Thyago C Vilhena, Rafaela C Santos Trindade, Márlia Regina C Ferreira, Sandro Percário, Luciana F Soares, Washington Luiz A Pereira, Geraldo C Brandão, Alaíde B Oliveira, Maria F Dolabela, Flávio De Vasconcelos

**Affiliations:** Programa de Pós-graduação em Ciências Farmacêuticas, Instituto de Ciências da Saúde (ICS), Universidade Federal do Pará (UFPA), Rua Augusto Corrêa 1, 68075-110 Belém, PA Brazil; Departamento de Botânica, Museu Paraense Emilio Goeldi, Belém, PA Brazil; Laboratório de Pesquisa em Estresse Oxidativo, Instituto de Ciências Biológicas (ICB), Universidade Federal do Pará (UFPA), Belém, PA Brazil; Faculdade de Farmácia, Universidade Federal de Minas Gerais, Belo Horizonte, MG Brazil; Departamento de Patologia Veterinária e Medicina Preventiva, Instituto de Saúde e Produção Animal (ISPA), Universidade Federal Rural da Amazônia, Belém, PA Brazil; Programa de Pós-graduação em Inovação Farmacêutica, Instituto de Ciências da Saúde (ICS), Universidade Federal do Pará (UFPA), Belém, PA Brazil; US Centers for Disease Control and Prevention (CDC), Atlanta, GA USA; Escola de Farmácia, Universidade Federal de Ouro Preto, Ouro Preto, MG Brazil

**Keywords:** *Himatanthus articulatus*, Malaria, Toxicity, Plumieride, Oxidative stress

## Abstract

**Background:**

*Plasmodium falciparum* has become resistant to some of the available drugs. Several plant species are used for the treatment of malaria, such as *Himatanthus articulatus* in parts of Brazil. The present paper reports the phyto-chemistry, the anti-plasmodial and anti-malarial activity, as well as the toxicity of *H. articulatus.*

**Methods:**

Ethanol and dichloromethane extracts were obtained from the powder of stem barks of *H. articulatus* and later fractionated and analysed. The anti-plasmodial activity was assessed against a chloroquine resistant strain *P. falciparum* (W2) in vitro, whilst *in vivo* anti-malarial activity against *Plasmodium berghei* (ANKA strain) was tested in mice, evaluating the role of oxidative stress (total antioxidant capacity - TEAC; lipid peroxidation – TBARS, and nitrites and nitrates - NN). In addition, cytotoxicity was evaluated using the HepG2 A16 cell-line. The acute oral and sub-chronic toxicity of the ethanol extract were evaluated in both male and female mice.

**Results:**

Plumieride was isolated from the ethyl acetate fraction of ethanol extract, Only the dichloromethane extract was active against clone W2. Nevertheless, both extracts reduced parasitaemia in *P. berghei-*infected mice. Besides, a significant reduction in pulmonary and cerebral levels of NN (nitrites and nitrates) was found, as well as in pulmonary TBARS, indicating a reduced oxidative damage to these organs. The ethanol extract showed low cytotoxicity to HepG2 A16 cells in the concentrations used. No significant changes were observed in the *in vivo* toxicity studies.

**Conclusions:**

The ethanol extract of *H. articulatus* proved to be promising as anti-malarial medicine and showed low toxicity.

## Background

The World Malaria Report featured that 207 million cases of malaria occurred worldwide in 2012 with 627.000 deaths [1]. Malaria is found in more than 100 countries, mainly in tropical regions of the world, including Africa, Asia, Central and South America. In Brazil, almost all cases occur in the Legal Amazon [2]. In Brazil, between the years 2000 to 2011 there was a reduction in the number of malaria cases. In 2011 the number of reported cases was 20% lower than previous years [3].

The first attempts for a specific treatment of malaria date back to the early 18th century, with the use of Cinchona tree bark; quinine was isolated as the active ingredient in 1820 [4]. The extensive use of anti-malarial drugs has imposed a tremendous selective pressure on the parasites, leading to the emergence of resistance, particularly in the case of *Plasmodium falciparum* [5]. Resistance of *P. falciparum* to quinine was reported after 278 years of clinical use, while for the majority of anti-malarial drugs, such as proguanil, sulphadoxine-pyrimethamine and atovaquone, resistance was reported much earlier; in the case of chloroquine and mefloquine, resistance was described after only 10 years of clinical use [6].

New anti-malarial drugs are urgently needed. The candidate drugs ought to be active against both chloroquine- and artemisinin-resistant *P. falciparum* strains. It should provide a cure within a reasonable length of time (3 days or less), be safe, at low cost, and should be available in an appropriate formulation for oral use [7].

Investigation of plant-derived compounds is a valid strategy and this approach can take advantage from traditional knowledge of native populations. Indeed, natural products have yielded two of the most important drugs used to treat falciparum malaria so far, quinine and artemisinins [8]. *Himatanthus articulatus* is in popular use in Brazil for the treatment of several diseases, including skin infections, helminth infestations, gastric diseases, such as peptic ulcers and gastritis [9], tumours [10], syphilis [11], as a cough medicine [12], and as an anti-inflammatory and analgesic [13]. It has been used against malaria [14,15], but this activity does require validation studies.

Popularly known in Brazil as sucuuba, janaguba or sucuba, *H. articulatus* is found in the South America, including Panama, Colombia, Peru, Venezuela, Guyana, Suriname, French Guyana and in the Brazilian Amazon and the Atlantic Forest [12]. The species name *H. articulatus* (Apocynaceae) is synonymous to *Himatanthus rigidus*, *Plumeria articulata* Vahl, *Plumeria drastica*, *Plumeria microcalyx* and *Plumeria sucuuba* [16,17]. It is a perennial, heliophytic, selective, xerophytic and secondary plant that inhabits sandy or mixed soils. Its trees can reach 10 to 20 metres in height, present substantial trunks and broken bark, simple and alternate spiral leaves with glabrous coriaceous and entire margins, white flowers of yellow bell-shaped bases, phallic fruits, green colour when immature and dark brown when mature [18].

**Figure 1:**
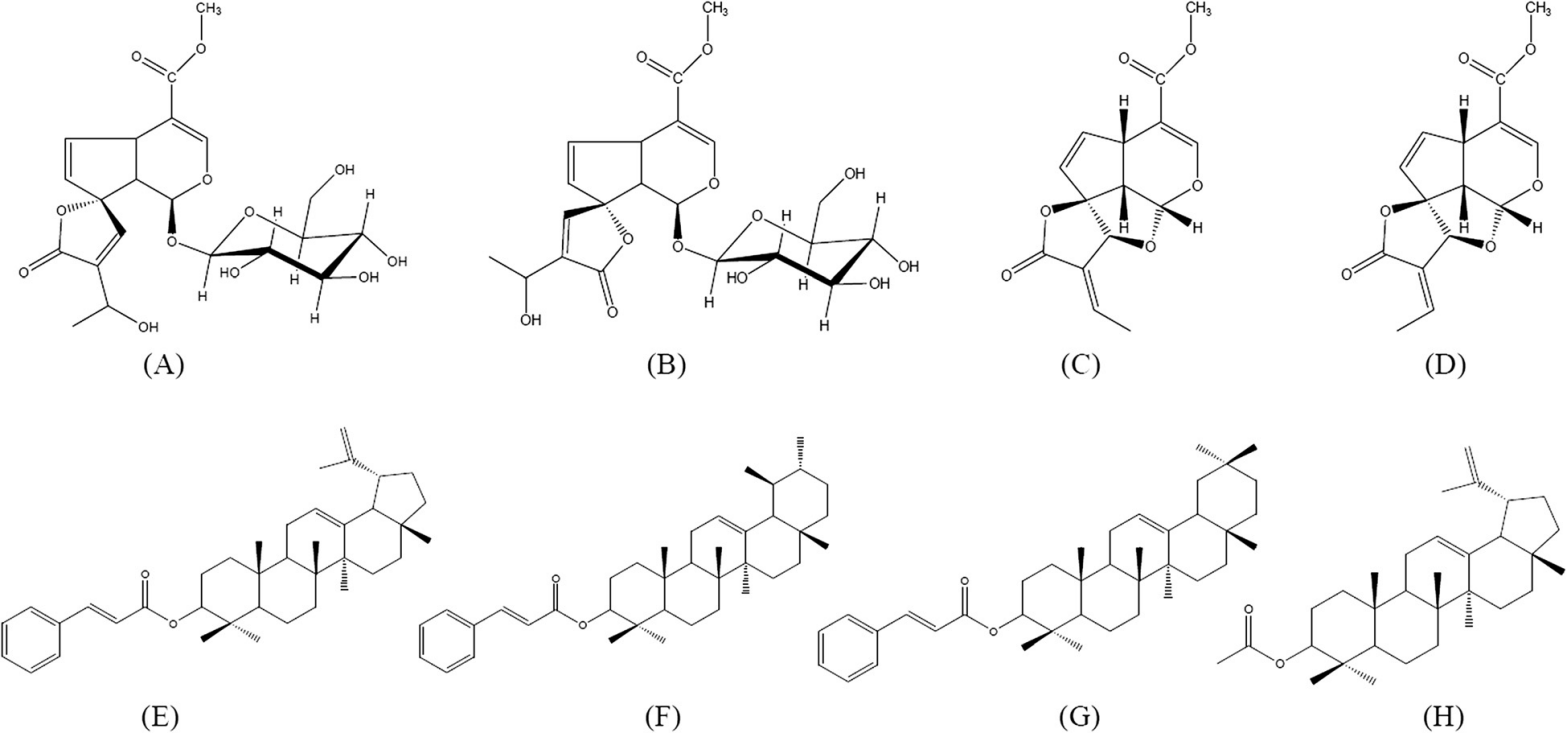
**Chemical structure of compouds occurring in** ***Himatanthus articulatus*** **.** **(A)** plumieride, **(B)** isoplumieride, **(C)** plumericin, **(D)** isoplumericin, **(E)** lupeol cinnamate, **(F)** α-amyrin cinnamate, **(G)** β-amyrin cinnamate, **(H)** lupeol acetate.

Several iridoids have already been isolated from this species: plumieride (Figure 1A), isoplumieride (Figure 1B), plumericin (Figure 1C) and isoplumericin (Figure 1D). In addition, also been isolated the triterpenes lupeol cinnamate (Figure 1E), α-amyrin cinnamate (Figure 1F), β-amyrin cinnamate (Figure 1G), and lupeol acetate (Figure 1H) [19,20].

Despite the broad popular use of *H. articulatus* bark and latex for the treatment of malaria [9,10], and the promising results described for terpenes [21,22], there is a lack of validation of this activity for this species. A single study has so far evaluated the anti-malarial activity against a chloroquine sensitive clone of *P. falciparum* (3D7), with no activity observed for the ethanol extract obtained from the cortex [10].

The present study describes, for the first time, the anti-plasmodial activity of *H. articulatus* against a chloroquine resistant clone of *P. falciparum* (W2), as well as the anti-malarial activity in *Plasmodium berghei-*infected mice. Moreover, it describes the results obtained in phytochemical studies and cytotoxicity, acute oral and sub-chronic toxicity. This study also reports changes in oxidative stress caused in *Plasmodium berghei*-infected mice.

## Methods

### Plant material and phytochemical studies

Stem barks from *H. articulatus* were collected in Altamira city, state of Pará, Brazil (S 01°10’86” W 41°53’51.6”), in the Brazilian Amazon. The dried specimen was deposited in the Museum Paraense Emilio Goeldi (voucher specimen: MG-206619). Subsequently, the barks were washed and dried in an oven with air vent, and triturated in a knife-mill.

The powder (1.0 kg) was submitted to percolation with ethanol, followed by concentration in a rotary evaporator and lyophilization obtaining ethanol extract (168.2 g). Another part from *H. articulatus* stem bark powder (100 g) was submitted to percolation with hydrochloric acid (1N). The resulting acid solution was alkalized to pH 9 with ammonium hydroxide, affording the partition with dichloromethane. This solution was concentrated in a rotatory evaporator, obtaining the dichloromethane extract (0.32 g) [23].

The ethanol extract (20.00 g) was solubilized with dichloromethane and subjected to a reflux system (20 min). Then filtered, and the precipitate was subjected to a procedure similar to ethyl acetate and methanol [24]. The solutions were concentrated in a rotary evaporator, and it was obtained dichloromethane (2.615 g), ethyl acetate (5.38 g) and methanol (9.98 g) fractions. The ethyl acetate fraction (3.50 g) was fractionated by silica gel column (60.0 × 2.5 cm) and eluted with solvents and mixture of these at increasing polarities (hexane, dichloromethane, ethyl acetate and methanol). Fraction 6 (3.2 g) was later fractionated in chromatographic column with silica gel (80 × 2 cm) with solvents at increasing polarities. Sub-fraction 6-8 (1.01 g of plumieride) underwent spectroscopic analyses (NMR, LC-UV-MS).

The ethanol extract, fractions and plumieride underwent evaluation by high performance liquid chromatography with diode array detector (HPLC-DAD, Waters mod. 2695, USA), C-18 reverse phase column (5 μm, 125 × 45 mm, LiChrocart 125-4, Merck, Germany), at 40°C, flow rate of 1mL/min, wavelengths scanning from 220 to 400nm. As mobile phase, a linear gradient of 5% (acetonitrile) and 95% (aqueous solution of phosphoric acid 0.1% v/v) at time 0 and 65 min with 95% (acetonitrile) and 5% (aqueous solution of phosphoric acid 0.1% v/v) were used. For the dichloromethane fraction, the initial gradient was acetonitrile 90% and aqueous solution 10% of phosphoric acid 0.1% v/v, 10 to 30 minutes, followed by 80% of acetonitrile and 20% aqueous solution of phosphoric acid 0.1% v/v from 30 to 32 minutes. Finally, 50% of acetonitrile and 50% aqueous solution of phosphoric acid 0.1% v/v were used henceforth.

The following spectroscopy methods were used for structural identification: Ultra High Performance Liquid Chromatography coupled to UV and Mass Spectrometry by Electron Spray Ionization (UPLC-PDA-MS/ESI Acquity H-Class Core System® Waters, USA), ^1^H NMR, ^13^C NMR and DEPT 135 (Bruker NMR spectrometer Advance DPX 200, Bruker, USA). The positive mode was used to obtain electron spray ionization mass spectrometry (Acquity H-Class Core System® Waters, USA), with capillary voltage of 3.5 eV, the cone 60eV, CSH130 C-18 column (particles of 1.7 μm, 50 × 3 mm), flow of 0.3 mL/min, temperature of 40°C and UV detection between 220 and 400 nm. As mobile phase, a linear gradient was used, in which the initial time contained aqueous solution of formic acid 0.1% (A) and acetonitrile with formic acid 0.1% (B), in 10 min 5% of A and 95% B. The NMR used deuterated methanol (Merck, Germany).

### Cytotoxicity assay

The cell viability was determined by the MTT (3- (4,5-dimethyltrazol-2-yl)-2,5-diphenyl tetrazolium bromide) method according to Mosman [25]. HepG2 A16 cells (4x10^5^ cells/0.1 mL) were grown in RPMI-1640 (Roswell Park Memorial Institute 1640) medium (Sigma Aldrich®, USA), supplemented with 5% of foetal calf serum, kept in a 5% CO_2_ atmosphere at 37°C. The ethanol extract from *H. articulatus* was solubilized in RPMI-1640 and dimethyl sulphoxide (0.02%, v/v). After 24 h, the solution was added at different concentrations (in μg/mL: 1, 10, 100, 1000), followed by 24 hours of further incubation. The MTT (2.0 mg/mL) was added, followed by incubation at 37°C in an atmosphere of 5% CO_2_ for 4 hours. Dimethyl sulphoxide was added to each well, and the reactions were mixed to solubilize the formazan crystals. The optical density was determined at 570 nm and 630 nm to measure the signal and background, respectively (Stat Fax 2100 microplate reader, Awareness Technology, Inc., USA). The cell viability was expressed as a percentage of the control absorbance in the untreated cells after subtracting the appropriate background and the average cytotoxic concentration (CC_50_) was determined by linear regression.

### Evaluation of anti-plasmodial activity in vitro

*Plasmodium falciparum* (strain W2) was grown according to Trager and Jensen [26], synchronized with 5% sorbitol [27]. Evaluation of *in vitro* anti-malarial activity was performed by the quantification of parasitic enzyme lactate dehydrogenase (*p*LDH), [28,29]. The test used the trophozoite stage (parasitaemia of 2% and haematocrit of 1%) and different concentrations of test samples (in μg/mL: 50.00, 25.00, 12.50, 6.25 and 1.56). Normal not infected red blood cells (RBCs) were used as negative control and as positive control, parasitized- and non-treated RBCs. Chloroquine was used as a standard anti-malarial drug. After 48h of incubation under CO_2_, plates were frozen (twice) to promote cell lysis. Fifteen μL of lysate was added to Malstat reagent (100 μL) and NBT/FT (25 μL), followed by incubation (1h at 37°C) under light and subsequent reading at 540 nm. Cell viability (%) was calculated as the ratio between non-infected (100% viables) and infected (0% viables) RBCs. The IC_50_ was determined by means of linear regression curve.

### Evaluation of acute oral and subchronic toxicity

Male (n = 8) and female (n = 8, nulliparous and non-pregnant) albino Swiss mice, weighing 25-27g, obtained from the animal facility of the Instituto Evandro Chagas, (IEC), Ananindeua, Pará, Brazil, were kept in the vivarium of the Faculty of Pharmacy (UFPA) under controlled temperature and humidity, light and dark cycle of 12 h each, with pelleted food and filtered tap water *ad libitum*. They were daily submitted, weighed, and assessed for food and water intake. At the end of the experiment, animals were anesthetized and euthanized, samples of blood were drawn and target organs were removed for anatomo- and histopathological evaluation (liver, kidneys, pancreas, brain and heart) [30,31]. The following laboratory tests (in sub-chronic test) were performed: complete blood count, liver function tests, total proteins, albumin, uric acid, urea, cholesterol, triglycerides [31] and hepatic lipid peroxidation (TBARS method).

For the acute oral toxicity test, it was used the Fixed Procedure Test, according to the guideline from OECD [30], with minor modifications. The animals were submitted to treatment by a single dose (5,000 mg/kg; gavage) and were evaluated by the Hippocratic test during 14 days. For the sub-chronic toxicity test, animals were treated with extract (200 mg/kg and 100 mg/kg each group) for 38 days and a satellite group was maintained for a further 14 days without treatment [31]. All procedures were in accordance to the ethical principles of Animal Experimentation, and the study was approved by the Ethics Committee (CEPAN /IEC/SVS/MS) under report number 045/2009.

### Experimental in vivo malaria

The anti-malarial activity was performed as established by Peters [32], with modifications. Swiss albino male mice (n = 10) were 10 days orally pre-treated with the ethanol extract (200 mg/kg), followed by subsequent infection with *P. berghei*. New treatment (200 mg/kg) was performed for 5 days. On the 5^th^ day after the infection, blood samples were drawn, animals were anesthetized for euthanasia and brain and lungs were removed for evaluation of TEAC (Trolox® Equivalent Antioxidant Capacity), NN (Nitrite and Nitrate) and TBARS (Thiobarbituric Acid Reactive Substances). Animals (n = 10) treated with filtered tap water under the same conditions as their counterparts in the treated group were used as a negative control.

Determination of the TEAC was performed according to Miller *et al.* [33]. Lipid peroxidation was assessed by the measurement of the TBARS, according to Percário *et al.* [34]. The levels of NN were assayed by the Griess method [35], using the nitrate/nitrite colourimetric assay kit (Cayman Chemical Co., USA)

### Statistical analysis

For cytotoxicity and anti-plasmodial assay it was used linear regression. For acute oral toxicity, oxidative parameters and antioxidant defenses it was applied the Student *t*-test for non-paired data, with a significance level set at *p* < 0.05. For sub-chronic toxicity it was used analysis of variance (ANOVA), followed by Tukey's post hoc test (*p* < 0.05).

## Results and discussion

### Phytochemical analysis

**Figure 2:**
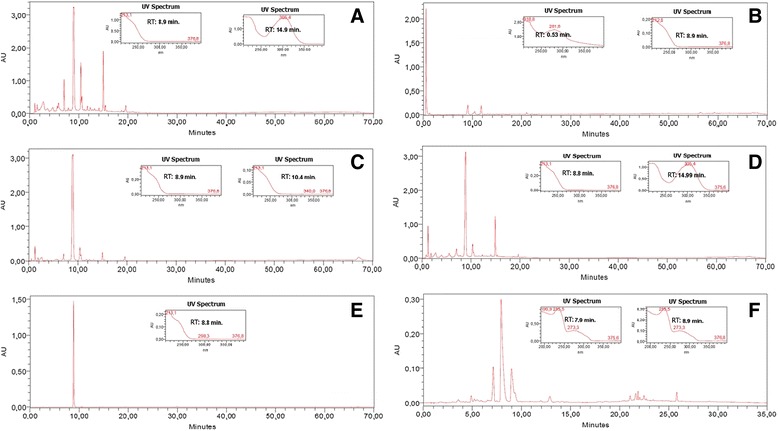
**Chromatograms profiles:** **A:** **EEHa (ethanol extract from** ***Himatanthus articulatus*** **stem barks);** **B:** **FrDCM (dichloromethane fraccion** **obtained from ethanol extract of** ***Himatanthus articulatus*** **stem barks);** **C:** **FrAcOET (ethylacetate fraction obtained from ethanol extract of** ***Himatanthus articulatus*** **stem barks);** **D:** **FrMeOH (methanol fraction obtained from ethanol extract of** ***Himatanthus articulatus*** **stem barks);** **E:** **PLUMIERIDE (iridoid compound isoled from FrAcOET)** ***;*** **F: DCME Ha (dichloromethane extract obtainded from** ***Himatanthus articulatus*** **stem barks).**

**Figure 3:**
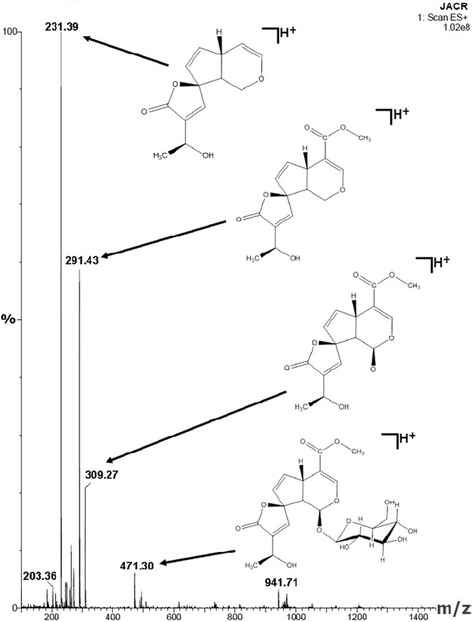
**Mass spectrum of plumieride registered online by UPLC-UV-MS/ESI showing pseudomelecular ion and fragments derived from it.**

The lyophilized ethanol extract from *H. articulatus* stem barks was fractionated by re-extraction under reflux yielding three fractions: the dichloromethane, the ethyl acetate and the methanol fractions (Table 1). The ethyl acetate fraction presented an HPLC-DAD profile (Figure 2C) suggestive of iridoids and, therefore, it was submitted to further fractionation affording sub-fraction 6-8 which was spectroscopically (UV, ^1^H NMR,^13^C NMR, DEPT 135 and MS) characterized as plumieride (Table 2 and Figure 3), the major iridoid found in this plant species [19,20,36].

**Table 1:** **Yield and anti-plasmodial activity of samples**

**Samples**	**Yield (%)**	**Anti-plasmodial activity IC**_**50**_ **± SD (μg/mL)**
EEHa	16.8	>50.00
FrDCM	13.1	>50.00
FrAcOET	26.9	>50.00
FrMeOH	49.9	>50.00
DCME Ha	0.2	22.90 ± 0.20
PLUMIERIDE	24.3	>50.00
Cloroquine	-	0.15 ± 0.06

EEHa-ethanol extract from *Himatanthus articulatus* stem bark; FrDCM- dichloromethane fraction obtained from ethanol extract of *Himatanthus articulatus* bark; FrAcOET- ethylacetate fraction obtained from ethanol extract of *Himatanthus articulatus* bark; FrMeOH-methanol fraction obtained from ethanol extract of *Himatanthus articulatus* bark; DCME Ha - dichloromethane extract obtained from *Himatanthus articulatus* bark powder; PLUMIERIDE-iridoid compound isolated from FrAcOET; Cloroquine-positive control.

**Table 2:** ^**1**^ **H and** ^**13**^ **C data for plumieride**

**Position**	**δ**_**H**_ **ppm (200 MHz)**		**δ**_**C**_ **ppm (200 MHz)**	
	**Presentwork**	**BARRETO** ***et al*** **., 2007**	**Present work**	**BARRETO** ***et al*** **., 2007**
1	5.3 (d)	5.5 (d)	94.2	93.5
3	7.5 (s)	7.6 (s)	152.6	152.7
4	-	-	111.1	111.1
5	-	-	40.4	38.8
6	6.4 (dd)	6.7 (dd)	141.5	143.3
7	5.5 (dd)	5.7 (dd)	130.0	129.1
8	-	-	97.9	97.6
9	3.2 (dd)	3.2 (dd)	50.6	46.4
10	7.4 (s)	7.6 (s)	150.3	152.7
11	-	-	138.6	139.3
12	-	-	172.8	174.6
13	-	-	63.6	63.6
14	1.4 (d)	1.6 (d)	22.5	22.4
15	-	-	168.5	170.4
16	3.7 (s)	3.9 (s)	52.1	53.6
1’	4.7 (d)	4.9 (d)	100.1	99.8
2’	2.9 (m)	3.9 (m)	74.7	74.3
3’	3.5 (m)	3.4 (m)	77.8	77.3
4’	4.5 (dd)	4.0 (m)	71.3	71.2
5’	3.8 (m)	3.6 (m)	78.9	78.0
6’	3.4 (m)	4.0 (m)	62.6	62.4

d-doublet, dd-double doublet, s-singlet, m-multiplet.

HPLC-DAD analysis of ethanol extract and fractions (Figure 2) showed the presence of peaks of compounds with high polarity. In all samples, except for the alkaloid fraction (Figure 2F), there is a predominant peak (retention time = 8.9 minutes) and an UV spectrum suggestive of iridoid chromophore [37]. This hypothesis was strengthened by injection of plumieride whose retention time and UV spectra were identical (Figures 2A, C, D and E). Due to low yield of fractions and plumieride, *in vivo* studies were not possible to be performed.

*Himatanthus articulatus* belongs to the Apocynaceae family, which is known to produce alkaloids [38]. However, previous studies have not yet described the isolation of alkaloids in this species [20,39]. Possibly, this class of natural products is a minor constituent in *H. articulatus*, if present at all. To address this question, a selective extraction for alkaloids was performed. The resulting dichloromethane extract showed three major peaks in the HPLC-DAD (Figure 2F). The UV spectrum of the most intense one showed λ_max_ = 235 and 273 nm. Due to the low yield (0.16%) of this extract no compound could be isolated. However, analyses by UPLC-UV-MS/ESI have shown that as the major constituent, showing a peak at RT = 7.91 min (52.83% area; Figure 2F) and a pseudo molecular ion [M + 1] = 329 u. Moreover, due to the low quantity of dichloromethane extract available, *in vivo* studies were not performed.

The chromatography study showed chemical similarity between the ethanol extract and its fractions (Figure 2). Currently, animal testing should be conducted in compliance with the principle of the 3Rs (reduction, refinement and replacement) [40]. As the ethanol extract has chemical similarities with all its fractions, it was decided to carry out *in vivo* studies with the ethanol extract only. Thus, there was a significant reduction of the number of animals used.

### Anti-malarial activity

All samples were submitted to anti-plasmodial activity evaluation by the pLDH method [28,29]. The ethanol extract, its fractions and plumieride were inactive against the chloroquine-resistant *P. falciparum* W2 clone (Table 1). Another iridoid (8-*THE-*acetylharpagide) also proved to be inactive against chloroquine-sensitive *P. falciparum* [41]. Collectively, these results suggest iridoid-rich extracts are inactive. Nevertheless, *in vivo* studies are still needed and deserve to be investigated. By contrast, the dichloromethane extract proved to be active against this clone (IC_50_ = 22.9 ± 0.2 μg/mL; Table 1).

The barks of *H. articulatus* are widely used for malaria treatment in the Amazon traditional medicine [9], suggesting the hypothesis that this species might interfere in the pathogenesis of the disease. Therefore, the ethanol extract was subjected to *in vivo* studies against *P. berghei*-infected mice when parasitaemia and oxidative parameters were evaluated (TBARS, TEAC and NN).

Further, contrarily to the *in vitro* study, the ethanol extract significantly reduced parasitaemia by 35.4% at a dose of 200 mg/kg. Plant extracts in which the reduction of parasitaemia is greater than 30% are considered active [42]. Several factors may be influencing this divergence of results amongst them: differences between the parasites used in the studies, or perhaps the extract contains a substance that requires prior hepatic metabolism to become active [43].

In addition, the mechanisms that trigger the respiratory distress present in pulmonary malaria are multifactorial. There is an evidence of the involvement of free radicals affecting cell membranes, attacking the endothelium and changing the vascular permeability [44]. An important mediator of this process seems to be nitric oxide [45].

Furthermore, Pino *et al.* [46] observed that the adhesion of parasitized erythrocytes to human pulmonary endothelium may be regulated by TNF, cytokine that induces the expression of iNOS enzymes, suggesting that the inhibition of NO synthesis may indeed protect pulmonary endothelial cells. Additionally, Dimmeler *et al.* [47] observed that iNOS inhibition by N-monomethyl-L-arginine (L-NMMA) impairs TNF induced endothelial cells apoptosis and, therefore, might be a protective mechanism.

In the present study, a significant decrease in the levels of brain and pulmonary nitrite and nitrate was observed (Table 3). These data suggest that the ethanol extract of *H. articulates* plays an inhibitor role upon NO synthesis and/or decreases NO concentration by directly scavenging it, thus potentially protecting the respiratory epithelium and reducing the severity of the respiratory distress to the animals.

**Table 3:** **Oxidative stress parameters of mice from both groups**

**Groups**	**NN (μM)**		**TBARS (μg/L)**		**TEAC (mM/L)**	
	**Brain**	**Lung**	**Brain**	**Lung**	**Brain**	**Lung**
**Infected**	507.3 ± 52.2	408.8 ± 49.1	224.8 ± 66.2	86.9 ± 40.5	20.0 ± 3.4	11.0 ± 3.3
**EEHa**	315.8 ± 57.0*	257.8 ± 48.0*	258.2 ± 57.7^NS^	38.0 ± 16.2*	21.5 ± 5.2^NS^	6.1 ± 9.6^NS^

Values are presented as mean ± standard deviation (n = 10 animals). Infected Group*: Plasmodium berghei*-infected mice; EEHa: *Plasmodium berghei*-infected mice and treated with EEHa (ethanol extract from *Himatanthus articulatus* stem bark) 200 mg/Kg, orally. NN: nitrite/nitrate; TBARS: thiobarbituric acid reactive substances; TEAC: Trolox® Equivalent Antioxidant Capacity. *p < 0.05 *versus* Infected Group. ^NS^Non-significant *versus* Infected Group. *Student’s.* “t” Test.

In parallel, the TBARS assesses the intensity of lipid peroxidation, which contributes to the inflammatory reaction due to the activation of pro-inflammatory cytokines, such as lymphotoxin and TNF) and IL-8. The reduction in TBARS levels found in the lungs of infected- and treated-animals (Table 3) may indicate a reduction of pulmonary inflammation and, possibly, an additional effect of NO decreased synthesis or of antioxidants present in the extract. In fact, in spite of no differences in TEAC values were seen between groups, it is possible to state that the ethanol extract may have some antioxidant properties, as several iridoids are regarded as antioxidant molecules [48,49].

In relation to brain findings, despite the reduction of nitrite and nitrate levels, no differences for TBARS and TEAC were found between groups. These results may indicate that the blood brain barrier could prevent the passage of certain constituents of the extract. Another possibility lies on the high cholesterol content of neuronal cells, as they are suitable to intense lipid peroxidation, whose might have surpassed the potential antioxidant effects of the extract.

In summary, the ethanol extract of *H. articulatus* reduced parasitaemia in *P. berghei*-infected mice. This extract also significantly interfered in oxidative parameters related to the pathogenesis of malaria. These results leads us to the following thought: when a plant species, of broad popular use for malaria, produces negative results in *in vitro* assays, *in vivo* studies ought to be performed. Such *in vivo* experiments should ascertain both clinical and parasitological aspects of the disease. Only then is it possible to validate anti-malarial activity of a certain natural product.

### Toxicity studies

Due to significant anti-malarial activity of the ethanol extract, cytotoxicity, acute *in vivo* and sub-chronic toxicity evaluations were performed. The ethanol extract from *H. articulatus* showed low cytotoxicity to HepG2 A16 cells in the concentrations used (CC_50_ > 1000 μg/mL). Nevertheless, in addition to the assessment of toxicity in cell lines, it is important to evaluate the oral acute and sub-chronic toxicity of the active sample in mammals.

The group of mice acutely treated orally with a high dose of the extract (5,000 mg/kg) showed neither immediate, nor during the observation period (14 days) clinical changes. No deaths occurred in the period of the experiment and no anatomopathological changes were observed in the organs evaluated. Besides, the differences between the sexes did not influence toxicity results.

Another study evaluated the acute toxicity of the extract from leaves of *H. drasticus,* with the animals showing signs of excitability, agitation and increased respiratory rate (dose = 2,000 mg/kg) [50]. Nevertheless, neither histopathological changes, nor other signs of toxicity were observed, suggesting that species of the genus *Himatanthus* have low acute toxicity.

No clinical changes or in water/chow intake were observed in the groups treated with the ethanol extract of *H. articulatus*. However, significant changes were observed on haematological and biochemical parameters (Tables 4 and 5).

**Table 4:** **Hematological values of 38 days EEHa-treated mice**

**Hematological parameters**	**Males (n = 8)**				**Females (n = 8)**			
	**C**	**T200**	**T100**	**S**	**C**	**T200**	**T100**	**S**
**WBC (10** ^**3**^ **/mm** ^**3**^ **)**	6.0 ± 1.6	10.0 ± 2.8	10.0 ± 1.6	7.9 ± 3.0	4.6 ± 0.4	5.4 ± 1.5	6.3 ± 1.4	14.5 ± 2.1*
**RCB (10** ^**6**^ **/mm** ^**3**^ **)**	6.5 ± 2.3	7.4 ± 0.7	7.8 ± 0.5	7.8 ± 0.7	5.0 ± 0.6	5.1 ± 0.8	7.6 ± 0.3*	7.9 ± 0.5*
**Hb (g/dL)**	12.1 ± 3.9	11.8 ± 1.3	12.9 ± 0.6	12.9 ± 1.4	7.8 ± 1.1	8.3 ± 0.9	13.0 ± 0.7*	13.3 ± 0.9*
**Hem (%)**	35.8 ± 1.1	35.1 ± 2.6	39.2 ± 0.9	39.6 ± 4.0	24.0 ± 2.5	25.2 ± 3.2	39.3 ± 1.5*	40.8 ± 2.5*
**MCV (fL)**	48.6 ± 1.6	49.1 ± 0.7	49.4 ± 0.9	50.5 ± 0.6	48.3 ± 0.7	48.8 ± 1.4	52.0 ± 0.5*	51.5 ± 0.6*
**MCH (pg)**	16.5 ± 1.1	16.0 ± 0.6	16.4 ± 0.7	16.3 ± 0.4	15.8 ± 0.7	15.9 ± 0.6	16.9 ± 0.7	16.8 ± 0.3
**HCMC (g/dL)**	33.6 ± 1.1	32.4 ± 0.6	32.9 ± 0.5	32.4 ± 0.4	31.8 ± 0.5	32.7 ± 0.5	32.9 ± 0.7*	32.6 ± 0.5
**Plat (10** ^**3**^ **/mm** ^**3**^ **)**	244.5 ± 63.0	445.3 ± 74*	403.0 ± 53.7	400.0 ± 145.1	283.3 ± 7.8	207.0 ± 43.4	346.0 ± 49.5	403.3 ± 161

EEHa: Ethanol extract from *Himatanthus articulatus* stem bark; WBC- white blood cell count; RCB- red blood cell count; Hb- hemoglobin; Hem- hematocrit; MCV- mean corpuscular volume; HCM- mean corpuscular hemoglobin; CHCM- mean corpuscular hemoglobin concentration; Plat- platelets; C- control group; T200- group treated orally with EEHa (200 mg/Kg); T100- group treated orally with EEHa (100 mg/Kg); S- satellite group. *p < 0.05 *versus* control group.

**Table 5:** **Laboratory patterns of 38 days EEHa-treated mice**

**Laboratory Parameters**	**Males (n = 8)**				**Females (n = 8)**			
	**C**	**T200**	**T100**	**S**	**C**	**T200**	**T100**	**S**
**AST (UI/L)**	136.7 ± 52.3	111.0 ± 56.6	147.0 ± 59.6	82.2 ± 17	83.5 ± 6.4	124.0 ± 18.4 *	65.0 ± 32.4	88.3 ± 19.5
**ALT (UI/L)**	7.7 ± 2.9	8.2 ± 1.7	7.5 ± 1.3	8.2 ± 2.1	16.0 ± 1.4	7.0 ± 1.4*	6.0 ± 0.0 *	6.4 ± 0.9*
**ɣGT (UI/L)**	11.5 ± 10.6	4.0 ± 0.0	4.2 ± 0.4	4.0 ± 0.0	4.0 ± 0	4.3 ± 0.6	4.0 ± 0.0	4.0 ± 0.0
**Alb (g/dL)**	2.9 ± 0.4	2.3 ± 0.2 *	2.6 ± 0.1	2.4 ± 0.2	3.4 ± 0.9	2.4 ± 0.5	2.4 ± 0.4	2.4 ± 0.2
**TP (g/dL)**	6.4 ± 2,35	4.2 ± 0.2*	4.3 ± 0.1	4.5 ± 0.4	4.7 ± 0.6	3.6 ± 0.7	3.9 ± 0.3	4.7 ± 0.3
**TC (mg/mL)**	95.0 ± 15.4	83.7 ± 3.2	86.0 ± 4.2	86.7 ± 4.3*	99.3 ± 10.0	66.6 ± 7.6*	83.5 ± 5.8	67.3 ± 3.2*
**TGD (mg/mL)**	163.0 ± 31.1	205.7 ± 15.0*	185.3 ± 12.7	229.7 ± 2.5*	173.3 ± 17.8	132.5 ± 17.7	154.0 ± 12.1	163.8 ± 18,5

EEHa: Ethanol extract from *Himatanthus articulatus* stem bark; AST-aspartate aminotransferase; ALT-alanine aminotransferase; ɣGT- gamma glutamyl transferase; Alb- albumin; TP- total protein; TC- Total cholesterol; TGD- triglycerides; Ur- urea; UA- uric acid; C- control group; T200- group treated orally with EEHa (200 mg/Kg); T100- group treated orally with EEHa (100 mg/Kg); S- satellite group. *p < 0.05 *versus* control group.

In the present study, haematological alterations were observed only in females, even in the group treated with the lowest dose (Table 4). At a dose of 100 mg/kg in females, the following changes were observed: increases in red blood cell-count, haemoglobin, haematocrit, MCV and MCHC (Table 4). The elevations of MCV and MCHC suggest macrocytosis and this should be investigated. In addition, there was an increase in the number of platelets and leukocytes. However, at the highest dose, these changes were only observed in the satellite group (Table 4).

It is known that females are more vulnerable to adverse drug reactions, as well as its toxic effects. Perhaps, the hormones related to this gender change significantly the pharmacokinetic aspects. These changes, perhaps, might have contributed to the vulnerability to toxic hematological effects. The biochemical changes occurred in both genders, being that in males it was seen at the highest dose (200 mg/kg). The reduction of albumin and total protein may be related to the inhibition of hepatic protein synthesis. Nevertheless, this inhibition is possibly reversible, as it has not been observed in the satellite group (Table 5).

**Figure 4:**
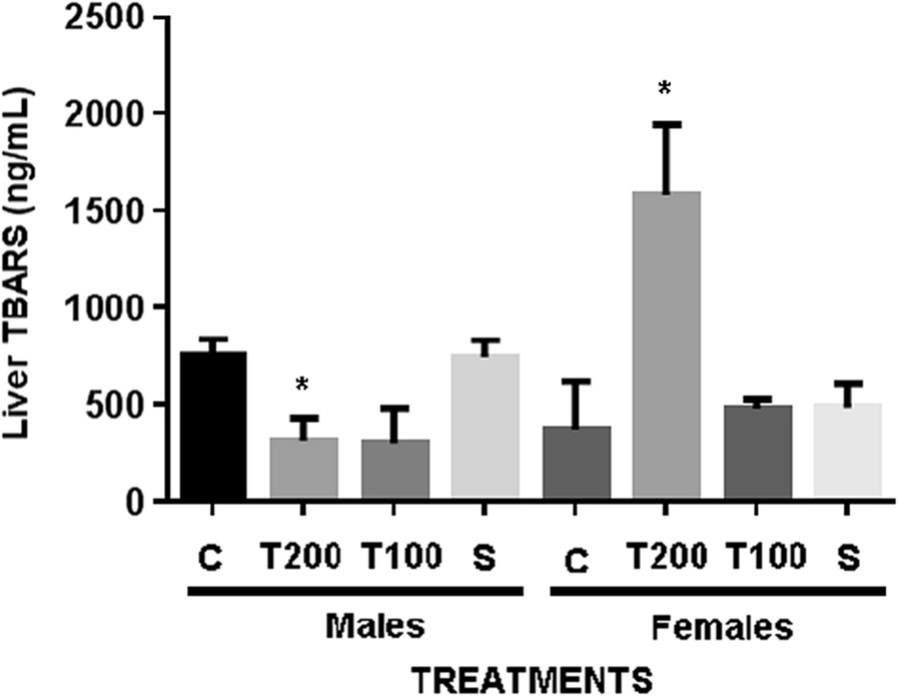
**Liver thiobarbituric acid reactive substances (TBARS) values from 38 days EEHa-treated mice.** C- control group; T200- group treated orally with EEHa (200 mg/Kg); T100- group treated orally with EEHa (100 mg/Kg); S- satellite group. *p < 0.05 *versus* control group (8 animals/group).

Other changes observed in males included: increase of triglycerides and a reduction in uric acid levels (Table 5). The increase of uric acid synthesis has been related to increased antioxidant activity. It is also a marker of the ischemia-reperfusion syndrome [51]. In the present study, the decrease of uric acid may suggest a lesser extent of ischemic events than in the control group. In the group of male animals, treated with 200 and 100 mg/kg of extract, a significant reduction of hepatic TBARS was observed. On the other hand, in the group of females TBARS levels were significantly increased (Figure 4). TBARS is a widely used lipid peroxidation marker and its increase can suggest high rate of cell membrane damage, while the reduction in male can signal the reduction of damage. As stated earlier, changes in pharmacokinetic properties, and in particular in the metabolism, might justify the differences in the responses amongst genders.

## Conclusion

An extract from *H. articulatus* bark containing plumieride as the major constituent. The ethanol extract from *H. articulatus* stem barks, although inactive *in vitro* (against *P. falciparum*), showed activity against *P. berghei* in an *in vivo* study. The ethanol extract may contain a substance that after metabolism becomes active. Another relevant fact found in the present study was the safety of this extract. The ethanol extract showed low cytotoxicity (IC_50_ > 1000 mg/mL), causing no significant clinical changes both in acute and sub-chronical administration. Besides, no histopathological changes were observed. Only biochemical and haematological reversible changes were observed. This study attested some anti-malarial activity of the *H. articulatus* extracts, as well as its safety, thus validating its popular use.
